# Transforming credit risk evaluation in digital lending from black box models to transparent decisions

**DOI:** 10.1038/s41598-026-51764-9

**Published:** 2026-05-13

**Authors:** Anber Abraheem Shlash Mohammad, Suleiman Ibrahim Mohammad, Asokan Vasudevan, S. M. Ferdous Azam, Lakshmi Sevukamoorthy, Manoranjan Parhi, M. Ugarthi Shankalia, Zaeid Ajsan Salami

**Affiliations:** 1 Digital Marketing Department, Faculty of Administrative and Financial Sciences, University of Petra, Amman, Jordan; 2 Electronic Marketing and Social Media, Economic and Administrative Sciences, Zarqa University, Zarqa, Jordan; 3 Research Follower, INTI International University, 71800 Nilai, Negeri Sembilan Malaysia; 4 Faculty of Business and Communications, INTI International University, 71800 Nilai, Negeri Sembilan Malaysia; 5 Management and Science University, Shah Alam, Selangor Malaysia; 6 Department of Management, School of Management – PG, JAIN (Deemed to be University), Bangalore, Karnataka India; 7 Department of Computer Science and Engineering, Siksha ‘O’ Anusandhan (Deemed to be University), Bhubaneswar, Odisha 751030 India; 8 Department of LAW, Sathyabama Institute of Science and Technology, Chennai, Tamil Nadu India; 9 Department of Computers Techniques Engineering, College of Technical Engineering, Islamic University in Najaf, Najaf, Iraq

**Keywords:** Credit risk prediction, Digital lending, Hybrid machine learning, Nature-inspired optimization, Explainable artificial intelligence, Engineering, Mathematics and computing

## Abstract

Digital lending and alternative finance systems, particularly Buy Now, Pay Later (BNPL) services, have expanded access to credit but introduced new challenges for accurate and transparent credit risk assessment. Borrowers in these ecosystems often lack extensive financial histories, making it difficult for traditional scoring models to capture their financial, behavioral, and socioeconomic characteristics. In addition, many existing machine learning approaches operate as black-box models, limiting interpretability and raising concerns regarding regulatory compliance and trust. This study proposes an optimization-driven hybrid machine learning framework that integrates gradient boosting models with nature-inspired metaheuristic optimization to enhance both predictive performance and interpretability in credit risk assessment. The proposed approach incorporates systematic data preprocessing, handling of class imbalance, and feature engineering to extract meaningful patterns from a publicly available dataset of 1,000 loan applications with 16 predictive attributes. Hyperparameters of the predictive models are optimized through iterative refinement, enabling efficient exploration of the search space and improved generalization. To ensure transparency, the framework provides feature-level explanations that identify the most influential variables contributing to default prediction. The model is evaluated using multiple performance metrics, demonstrating improved stability and predictive capability across cross-validation folds. Unlike conventional black-box approaches, the proposed framework balances accuracy with interpretability, making it suitable for deployment in regulated financial environments. The findings demonstrate that combining gradient boosting with optimization techniques yields a robust and explainable solution for credit risk prediction. This study contributes to the advancement of interpretable artificial intelligence in digital lending by offering a practical and transparent modeling framework that supports reliable decision-making.

## Introduction

### Background

The rapid expansion of Buy Now, Pay Later (BNPL) and other financing models in e-commerce has changed the way customers make purchases, thus these methods have been credited with a “revolution” in customer purchase patterns. These financing options are the most convenient way to obtain a short-term loan for a purchase, especially when credit history is unknown or rarely exists ^1^. Typically, the majority of BNPL users do not fit the standard financial profiles; hence, it is very difficult to assess their ability to repay loans, which consequently raises credit risk for lenders. Traditional credit score algorithms are mainly based on loan repayments, credit card transactions, and banking activities; they exclude underbanked and unbanked customers from the financial system ^2,3^. Artificial intelligence (AI)- driven financial scoring models have been a strong contender in overcoming the limitations imposed by traditional methods ^4^. In AI-powered credit assessment methods, deep learning algorithms are used to understand not only financial but also behavioral data from a wider range of individuals, including social media activities, digital footprints, and transactional behaviors ^5,6^. These models are more effective in evaluating non-traditional kinds of borrowers since, unlike conventional methods, they can assess risk on a dynamic basis and thus can be updated with real-time data inputs ^7^. AI methods can discover creditworthy individuals whom traditional credit evaluation systems may overlook through the use of alternative data ^8,9^. This method, with its more precise risk predictions, not only facilitates financial inclusion but also reduces the number of default cases ^10^. The use of reinforcement learning to upgrade BNPL credit risk assessments has been presented by recent scholarly works as one of the future directions ^11^. Reinforcement learning achieves this by constantly absorbing new borrower behavior and accordingly updating risk predictions, thus AI models can make better credit approval decisions ^12^.

The integration of these approaches into BNPL systems thus lessens the chances of monetary losses by lenders through the assistance of improved loan structuring, tailored credit limitations, and real-time fraud detection ^13,14^. In addition, the use of machine learning and big data analytics in credit risk models not only speeds processing but also enables scaling, allowing lenders to expand their customer base while maintaining financial stability. This study suggests that when AI-powered credit risk assessment fills the voids left by conventional methods of credit evaluation, it can be a game-changer for BNPL financing ^15,16^. As AI becomes increasingly advanced, the integration of AI into financial decision-making processes might be the radical change that digital lending ecosystems are looking for ^17^. This change can drastically impact the inclusiveness and credit availability facet of the digital lending ecosystem, thus reshaping the whole ecosystem and becoming a revolutionary move for the future ^18^.

### Literature review

Artificial intelligence (AI) and machine learning (ML) are among the very few recent studies that have attracted attention because of their potential to raise the quality of financial decision-making and to make credit risk assessment more accurate through predictions.

#### Black-box machine learning and deep learning models

A thorough evaluation by Roy et al. ^19^ explained AI-powered methods that help credit-granting decision processes become not only more efficient but also fair to all groups of borrowers. Besides, their investigation indicates that variances in the places where models are set up and operated are resulting in a halt in data quality, openness, and legal constraints; however, the significant speed improvements at the same time reveal promising research directions. Chafale et al. ^20^ expanded the investigation of ML implementations in credit risk analysis by uncovering the ways in which data-driven models can enhance both scalability and prediction accuracy. These models provide tools for instantaneous evaluation and can adapt to the evolving behavior of the debtor by leveraging a wide range of datasets and features. While additional data and enhancements to the modeling method are still necessary, the authors emphasize that these kinds of systems can facilitate the implementation of fair lending norms and thus contribute to strengthening the financial system’s stability. Neural network models have shown to be very powerful instruments for credit evaluation. In their paper on credit risk in Filipino multifunctional cooperatives, Papa and Ricafort ^21^ showed that Artificial Neural Networks (ANN) outperformed traditional models, thus achieving 86% of accuracy and 90% of ROC AUC. Even though CNN had low recall but very good accuracy, the RNN also exhibited good performance. The paper presents a data-driven basis for improved credit risk assessment and highlights the potential of ANNs and RNNs in the cooperative lending sector. Soni et al. ^22^ experimented various machine learning algorithms such as Support Vector Machines (SVM), Random Forest, XGBoost, and Decision Trees. Decision Trees were chosen for their interpretability, Random Forest and XGBoost for their outstanding predictive performance, and SVM for their effectiveness even when scaling issues were present. The article, by identifying feature selection, data quality, and model transparency as the most challenging issues, suggests creating hybrid, interpretable AI models as the next step in the research.

The significance of machine learning in making fraud detection and credit risk assessment more efficient was emphasized by Muhindo et al. ^23^. Their study demonstrates increased prediction consistency with large, complex datasets by the use of advanced algorithms and detailed feature engineering and preprocessing.

#### Interpretable and explainable AI models

De Silva et al. ^1^ moved this area of research beyond the limits by employing human supervision in conjunction with AI models like SVM, Logistic Regression, and Decision Trees. They achieved a prediction accuracy of 99% while also keeping the system understandable and compliant with regulations. Zhou et al. ^2^ examined interpretable machine learning in supply chain finance by comparing CNN, LSSVM, Random Forest, and XGBoost. As per their results, XGBoost outperforms other models, and the SHAP analysis identified asset-liability ratio, cash ratio, and quick ratio as the most significant features. These interpretable models eliminate the issues of black-box methods and hence, they open the way for transparent, informed, and data-driven financial decisions. Together, these papers illustrate how ML and AI can drastically change the landscape of credit risk assessment by emphasizing factors such as accuracy, interpretability, and ethical decision-making, and also acknowledge that there are still issues of data quality, scalability, and model transparency that need to be further researched.

Table 1 presents a detailed summary of all the recent studies that have used ML and AI methods for financial risk assessment. It includes the exact methods used, such as neural networks, decision trees, support vector machines, and XGBoost, along with the sectors where they were applied, e.g., supply chain finance, consumer finance, and cooperative lending. The first three columns of the table depict the main points of each paper, which include predictive performance, interpretability, and insights into significant financial indicators. This table presents the current state of AI/ML applications in financial risk analysis, highlighting constraints and potential research directions to address issues related to data quality, model transparency, scalability, and regulatory compliance.

**Table 1:** Summary of the related studies related to the credit risk assessment in digital lending ecosystem.

References	Methods	Context	Key findings	Limitations/future directions
^19^	AI-driven techniques	Business and consumer financial data	Improved prediction accuracy; facilitates fairer lending decisions; highlights geographic differences	Persistent issues with data quality, model transparency, and regulatory compliance; need for further research
^20^	Machine learning, Python-based adaptive models	Diverse borrower datasets	ML enables scalable operations and accurate forecasts; supports real-time assessments; promotes ethical lending	Continuous improvement of data and modeling techniques required
^21^	ANN, RNN, CNN	Philippine multipurpose cooperatives	ANN achieved 86% accuracy, 90% ROC AUC; RNN performed well; CNN limited by recall	Need to optimize model choice for cooperative credit risk assessment
^22^	Decision Trees, Random Forest, XGBoost, SVM	Various financial datasets	Random Forest/XGBoost high predictive performance; Decision Trees highly interpretable; SVM effective but scaling issues	Feature selection, data quality, and interpretability remain challenges; hybrid and scalable models needed
^23^	Neural networks, feature engineering	Large, complex financial datasets	ML improves credit risk assessment and fraud detection; enhances the reliability of decisions	Continuous upgrade of algorithms needed; implementation challenges exist
^1^	SVM, Logistic Regression, Decision Trees	World Bank Findex, financial behaviors	Achieved 99% prediction accuracy; emphasizes human-in-the-loop approach; highlights debit card, mobile banking, and deposit patterns as key predictors	Need for automated real-time monitoring and interpretability for regulatory compliance
^2^	XGBoost, Random Forest, CNN, LSSVM, SHAP	Supply chain finance datasets	XGBoost outperformed others; asset-liability, cash, and quick ratios most significant; interpretable models enable transparent decisions	Black-box models lack transparency; interpretable approaches recommended for fair decision-making

#### Summary of existing challenges and research gap

Across the literature, several recurring challenges can be identified:

- Hyperparameter tuning inefficiency in complex models
- Model instability and sensitivity to parameter configurations
- Trade-off between interpretability and predictive performance
- Limited robustness under data imbalance and noisy conditions
- Lack of unified frameworks combining optimization and explainability

Although ensemble and interpretable models have been explored, few studies systematically integrate metaheuristic optimization techniques with gradient boosting models to simultaneously improve hyperparameter search efficiency, model stability, and interpretability.

### Objective of the study

The​‍​‌‍​‍‌ fundamental purpose of this research is to establish a dependable, understandable, and practically implementable framework for credit risk prediction by integrating state-of-the-art machine learning models with nature-inspired metaheuristic optimization techniques. The study employs 16 various financial, demographic, and behavioral indicators to reflect the complex factors that determine the creditworthiness of an individual. It is based on a dataset of 1,000 publicly available loan applications from the University of Santiago de Chile. This study addresses numerous significant holes in the existing body of knowledge. While models such as neural networks, gradient boosting, and ensemble methods have been demonstrated by previous studies to have a high predictive potential, the problem of model interpretability, scalability, hyperparameter tuning, and regulatory compliance still remains to be solved. Most traditional methods use grid/random search techniques that only mildly explore complex hyperparameter spaces, or rely on black-box models that are less transparent, lowering operational reliability. Besides that, the researchers hardly ever consider the interaction between interpretable models and metaheuristic optimization in performance improvement and generalization under the given constraints in the real world. The paper proposes three novel elements to fill these gaps:

1. *Methodological Synergy:* The framework combines the strengths of the individual components in a complementary manner—these strengths include computational efficiency, native handling of categorical features, interpretability, and global search optimization. The three machine learning models LightGBM, CatBoost, and Explainable Boosting Machine (EBM) are combined with the Brown-Bear Optimization Algorithm (BBOA) and Puma Optimizer (PO) to achieve a single, very accurate, and interpretable predictive system that is coherent.
2. *Enhanced Interpretability and Feature Insight:* Unlike typical black-box models, EBM’s feature-level transparency allows one to see clearly the factors that influence the risk of default. Besides being in line with the moral and regulatory standards of financial decision-making, this provides valuable insights for policy formation, risk reduction, and operational strategies.
3. *Operational and Real-World Applicability:* These are ensured through the iterative model parameter-optimization process: stable convergence, improved generalization, and resistance to data imbalance. Hence, the proposed system is not only a viable option for implementation in practice but also a dependable theoretical model in the area of finance, involving operational risk reduction, which is a function of stable and reliable forecasts.

Rather than claiming universal superiority, this work aims to demonstrate the practical benefits of integrating interpretable boosting models with metaheuristic optimization for credit risk prediction. The proposed approach provides empirical insights into how predictive accuracy, stability, and transparency can be jointly improved in data-driven lending systems.

**Fig. 1:**
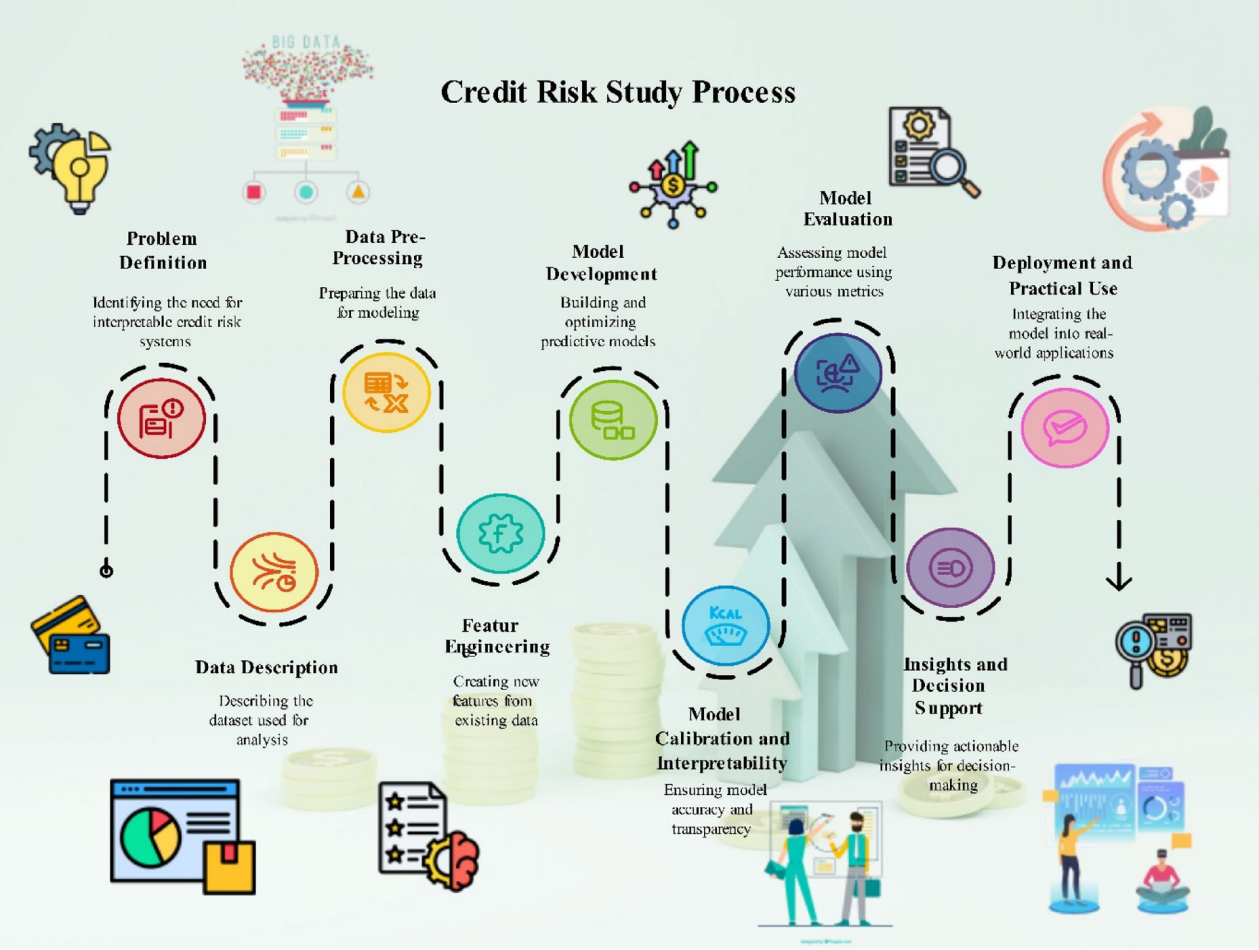
End-to-end framework outlining stages of the development of interpretable credit risk models.

An orderly, end-to-end approach to conducting a detailed credit-risk analysis is illustrated in Fig. 1, along with how interpretability and analytical rigor combine to deliver valuable, actionable insights. Problem Definition is the very first stage that recognizes the necessity of reliable and transparent credit-risk systems. Next, it moves to Data Pre-Processing and Data Description, which put the spotlight on the vital role of understanding, cleaning, and preparing datasets before any modeling. Feature engineering broadens the analytical ground by generating predictions from unprocessed data. After that, the sequence is handed over to Model Development, where predictive algorithms are developed and refined, and to Model Evaluation, which gauges performance by comparing against standard measures. Model calibration and interpretability facilitate accuracy and clarity to be combined, thus stakeholders can trust and understand model decisions. The final stages are Insights and Decision Support, Deployment, and Practical Use, embedding the model in operational environments, hence turning the analytical findings into strategic actions. In brief, the diagram represents a systematic, iterative journey that connects data, modeling, and decision-making into a single, seamless ecosystem for credit risk management.

## Materials and methods

### Data engineering

#### Dataset description

Although this study is motivated by Buy Now, Pay Later (BNPL) and digital lending ecosystems, the dataset used does not originate from a BNPL platform. Due to the proprietary nature of BNPL transaction data, a publicly available benchmark credit dataset with similar financial, demographic, and behavioral characteristics is adopted to validate the proposed methodology. The objective is to demonstrate a generalizable and interpretable modeling framework applicable to BNPL systems when real-world data are accessible. The dataset for the study consisted of 1,000 loan application records from the University of Santiago de Chile and is publicly available under the CC BY-NC-SA 4.0 license (https://www.kaggle.com/datasets/daniellopez01/credit-risk). The binary target variable (default) is from among 16 predictor variables, representing whether the loan applicant paid back the loan successfully (“no”) or was in default (“yes”). The predictors comprise a diverse set of financial, demographic, and behavioral features that are relevant to the credit risk assessment. For example, they include checking and savings balances, years of work, fraction of income allocated to loan repayment, existence of other credit, housing status, number of outstanding loans, age, type of job, requested loan amount, loan duration, and years of residence. Collectively, these features describe the applicants’ financial health, credit behavior, and socio-economic status, and they are very similar to the factors lenders assess when making real-world decisions.

Table 2 acts as a reference guide for the primary input variables that are used in a credit-related assessment model. Along with box plots that visually show the data distribution and central tendency, each variable is summarized by its lower and upper limits. Financial indicators such as checking balance, loan length, credit history, and amount, which show considerable variation, hence highlight their influence on risk assessment, are examples of these fluctuations. The intervals for demographic and stability-related parameters like age, years of work, and years of residency are a bit narrower. The model’s multifaceted viewpoint, further augmented by features such as residence, employment type, and outstanding loans, provides a comprehensive foundation for creditworthiness analysis.

**Table 2:** Core Risk Indicators are presented in an integrated manner, showing their limited values and statistical dispersion patterns.

Variable	Indicator		
	Lower bound	Upper bound	BOX plot
Checking balance	4	1	
Months loan duration	60	4	
Credit history	4	0	
Purpose	20	0	
Amount	15,857	250	
Savings balance	4	0	
Employment duration	4	0	
Percent of income	4	1	
Years at residence	4	1	
Age	75	19	
1 credit	2	0	
Housing	2	0	
Existing loans count	4	1	
Job	3	0	
Dependents	2	1	
Phone	1	0	
Default	1	0	

#### Pre-processing and feature engineering

In order to ensure data quality and suitability for machine learning models, several standard pre-processing steps were applied. Numerical variables such as age, loan amount, and income percentage were scaled to standardize their ranges, while categorical attributes were encoded using label encoding or one-hot encoding, depending on their cardinality. Missing values were handled based on variable type and empirical distribution. The term resampling in this study refers to dataset-level balancing considerations during model development rather than the application of synthetic oversampling techniques. No artificial samples were generated; instead, class distribution was monitored to ensure that preprocessing and modeling steps did not introduce unintended imbalance. Feature engineering focused on transforming existing variables to better reflect financial relationships inherent in the dataset. More specifically, the following derived features were constructed: (i) a loan-to-income ratio, calculated as the ratio of total loan amount to annual income, capturing repayment burden; (ii) an installment-to-income ratio, representing the proportion of periodic repayment obligations relative to borrower income; (iii) a credit utilization proxy, defined as the ratio of current credit usage to estimated credit capacity where available; and (iv) normalized loan size features obtained by scaling loan amount relative to population-level statistics (e.g., mean or standard deviation). In addition, interaction features between selected variables (e.g., income level × employment status) were explored to capture non-linear financial behavior patterns.

**Fig. 2:**
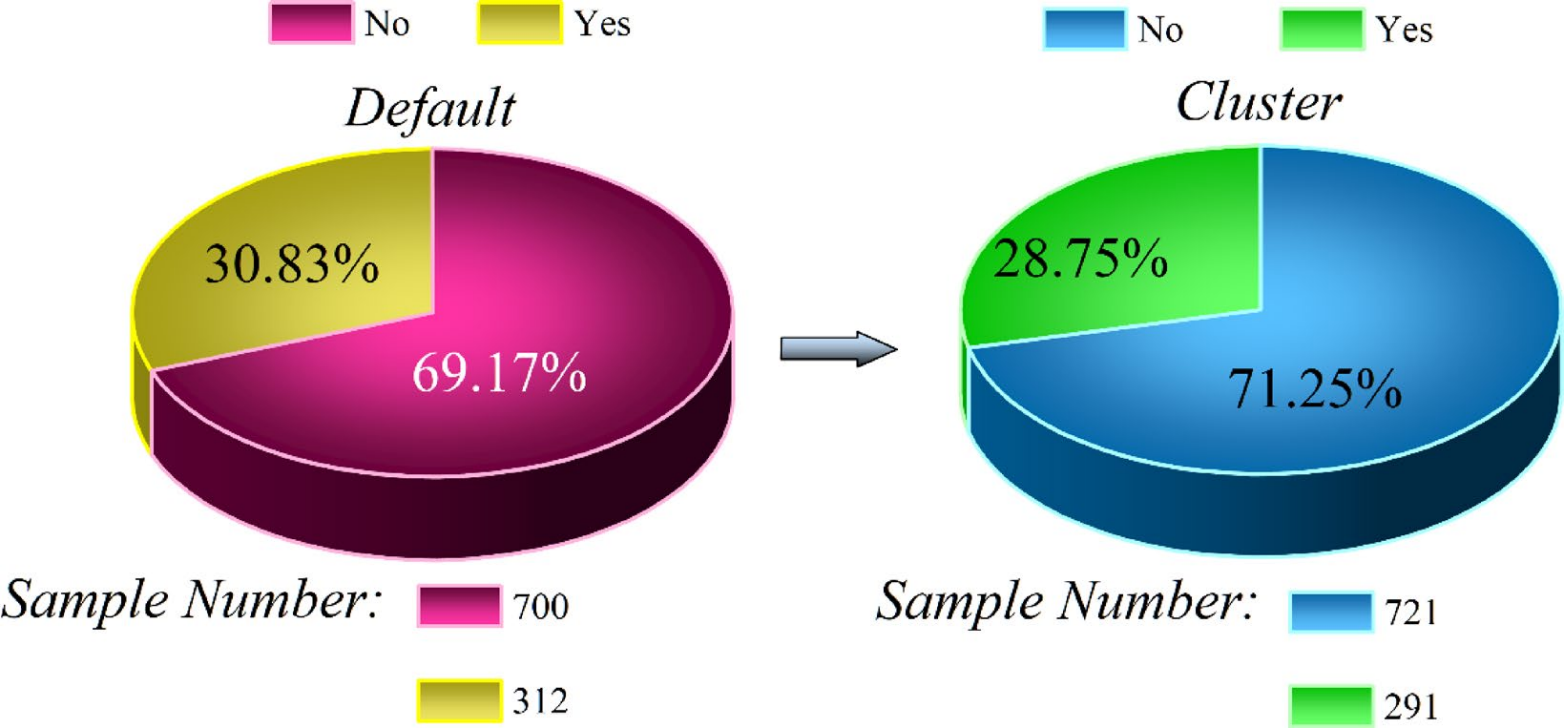
Cluster partitions generated by the K-Means algorithm, illustrating the grouping structure within the dataset.

Figure 2 illustrates the class distribution before and after clustering or classification operations, demonstrating that the overall balance between default and non-default classes was preserved. This confirms that the applied preprocessing and transformation steps maintained dataset stability and did not distort the underlying probability distribution, which is essential for fair and reliable credit-risk modeling.

In this study, K-means clustering was employed as an exploratory data analysis technique rather than as part of the predictive modeling pipeline. The objective was to identify latent group structures within the dataset based on borrower characteristics, such as financial capacity and loan attributes. By examining cluster composition, it was possible to assess whether naturally occurring groups exhibited distinct default patterns and to verify that preprocessing steps did not disrupt inherent data relationships. Importantly, the clustering results were not used as input features for the classification models, but instead served as a diagnostic tool to support data understanding and feature engineering decisions.

To address class imbalance, cost-sensitive learning was applied by assigning class weights inversely proportional to class frequencies. Specifically, the weight for the non-default class was set to 0.71, while the default class was assigned a weight of 1.67. These weights were incorporated into the training process of the machine learning models to increase sensitivity toward the minority class.

#### Dataset rationale

The multidimensional structure of this dataset and its similarity to datasets used in real-world lending situations make it a perfect tool for credit risk prediction research. By using a combination of financial, behavioral, and demographic features, a comprehensive depiction of applicant profiles and default trends is achieved. Financial indicators (such as loan amount, account balances, and income percentage) were combined with behavioral and demographic factors (such as credit history, job length, and residency stability) to produce a thorough depiction of applicant profiles and default trends. The dataset’s capacity to serve as a platform for empirical modeling is enhanced when there are strong correlations between key variables and default probability. Due to its size, feature diversity, and real-world relevance, it is a good benchmark for the development and evaluation of machine learning models in credit scoring environments such as microfinance, cooperative lending, and banking applications.

**Fig. 3:**
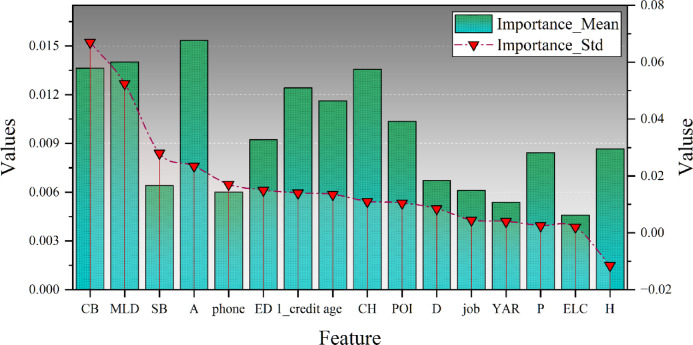
Results from permutation analysis used to assess feature influence on model performance.

Figure 3 displays the results of a permutation-based importance analysis, which involves systematically changing each feature to determine its contribution to prediction performance. The red points depict the variation of that effect over different permutations, whereas the turquoise bars represent the average effect of each feature on the model’s output. Individually, these measurements show how strongly and consistently each feature influences the system. This study has notable practical implications for real-world scenarios, a place where reliable and transparent decision-making is of core value. The features on the left side of the figure with a higher mean relevance are the main forces that drive the prediction process. Their strong influence implies that even a slight change in these factors in the real world may lead to a considerable change in the results, making them crucial points for enhanced data-quality initiatives, continuous monitoring, and process control. On the other hand, less important features reveal the context in small but significant ways. By identifying these lower-impact variables in the creation or management of large-scale systems, one may achieve resource efficiency, system simplicity, and dimensionality reduction. The permutation method’s analytical basis is in agreement with well-known concepts in physics and mathematics. From a mathematical point of view, the method is akin to standard sensitivity and perturbation analysis, which demonstrates that a function or system is stable under controlled variations of input. Here, the standard deviation stands for higher-order variability and interaction effects, while the mean importance represents the first-order impact of perturbation. Corresponding concepts can also be found in physics research on system dynamics and stability, where the degree of coupling between components is determined by small perturbations from equilibrium. The low-importance features, therefore, resemble weakly interacting components whose disturbances dissipate without system consequences, whereas the high-importance features are like variables with strong coupling, where perturbations spread throughout the system. The values of the figure provide an easy-to-grasp operational meaning. The higher bars of the graph indicate that a feature is used more heavily, and that it is also associated with a larger drop in prediction accuracy when randomization is applied to this feature. Larger standard deviations signal that the model is more sensitive in that region or there are interaction effects, as can be seen from the different impact among permutations. Those features with lower averages and smaller variances make modest, continuous contributions, thereby facilitating the improvement of forecasts.

### Rationale for model selection

#### Ensemble gradient boosting models (LightGBM, CatBoost, EBM)

Gradient boosting algorithms were the first choice as the main supervised learning models because of their demonstrated capability to deal with high-dimensional, nonlinear, and tabular financial datasets, which are standard in credit default prediction. Nevertheless, each model brings a different methodological strength:

- LightGBM provides a structure optimized for efficient histogram-based splitting and leaf-wise tree growth. These features allow it to scale to large datasets while maintaining high accuracy; thus, it is a perfect candidate for scenarios where computational limitations and real-time scoring are present. Moreover, the use of GPU acceleration can be exploited by LightGBM further to make extensive hyperparameter tuning and rapid experimentation more efficient ^24^, ^25^.
- CatBoost, on the other hand, was chosen to address the dataset’s categorical richness. A lot of financial attributes that are used in the prediction inherently have a categorical structure (e.g., job type, housing status, credit history categories). In CatBoost, the native handling of categorical features removes the need for a feature encoding scheme and, at the same time, avoids distortions of one-hot encodings ^26^. Ordered boosting improves generalization, and prediction shift is reduced, so CatBoost can be trusted in production scenarios more than other models ^27^.
- EBM is a transparent, GAM-based model that helps in understanding the two high-performance models. Normally, boosting is considered a “black box,” but EBM maintains the additive structure while augmenting it with learned pairwise interactions, allowing users to see and verify how features like income ratios or account balances are factors that increase the predicted risk ^28^. EBM was the model selected to help explain decision-making in the financial industry, a sector that is not only legally but also ethically required to make decisions in an accountable way ^29^.

#### Metaheuristic optimization algorithms (BBOA, PO)

To evaluate optimization-based methods for credit risk categorization, the study combines predictive models with two nature-inspired metaheuristic algorithms: the Brown Bear Optimization Algorithm (BBOA) and the Puma Optimizer (PO). The algorithms have two functional roles: Model hyperparameter optimization, where the combinatorial nature of boosting hyperparameters may make a typical grid or random search inefficient. Independent categorization capacity provides a different perspective on the solution landscape. In an effort to effectively dissect intricate, multimodal solution spaces, BBOA was integrated with its scent-signaling and movement-inspired search mechanisms, which give a smart way of balancing exploration and exploitation. This versatility is good for tuning models with many interacting parameters, such as gradient boosting ^30^. PO was selected because of its energetic hunting-inspired approach, which goes for the combination of the thorough exploration with the very focused exploitation ^31^. Nonlinear optimization is its strong suit, and in cases where traditional optimization would stall due to local optima, PO can break free, enabling it to be used to improve model configurations ^32^. By using these two opposite metaheuristics, the study not only compares boosting models but also asks whether biology-inspired optimization may be a significant approach to hyperparameter search and classification.

**Fig. 4:**
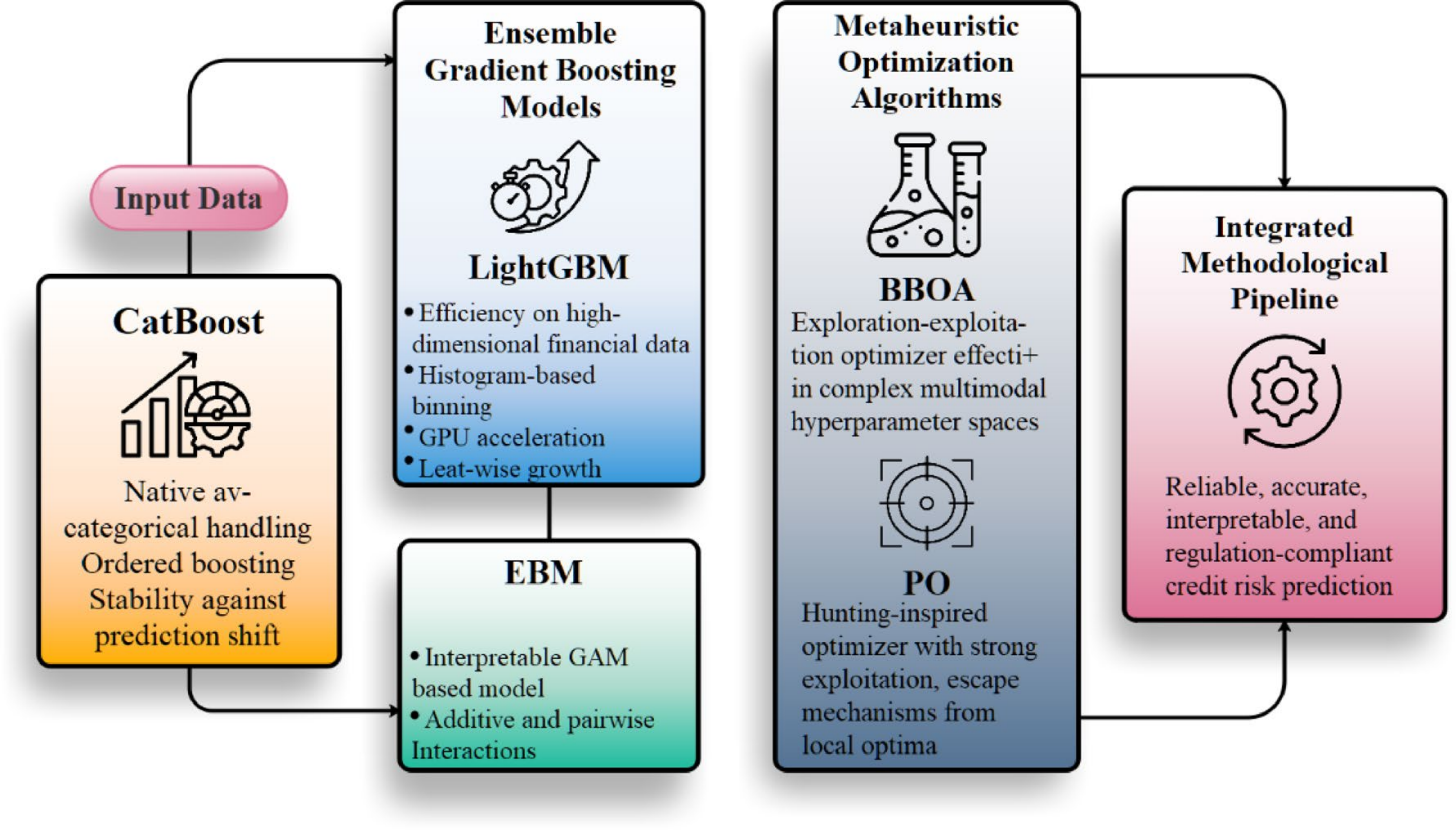
Gradient boosting models, together with metaheuristic optimization, are employed in an integrated pipeline to provide a high-accuracy, interpretable, and regulatory-compliant prediction framework.

The pipeline of Fig. 4 fuses state-of-the-art machine learning and optimization strategies to form a robust, interpretable prediction framework. On the one hand, LightGBM offers efficient training via histogram-based binning and leaf-wise growth, while on the other hand, CatBoost provides a good handling of categorical features. EBM brings in transparent, GAM-based modeling with pairwise and additive interactions. Metaheuristic optimizers like PO for intensive exploitation and escape from local optima and BBOA for efficient exploration of complex hyperparameter spaces are used to enhance these models. The final integrated system, thus, is capable of producing accurate, reliable, and interpretable outputs that ensure robust, transparent predictive performance across different scenarios, thereby being suitable for regulated, high-stakes decision contexts.

#### Synergistic methodological design

The credit risk prediction framework, using the credits, has combined LightGBM, CatBoost, EBM, BBOA, and PO to leverage complementary methodological capabilities. LightGBM, using leaf-wise tree growth, histogram-based binning, and GPU acceleration, provides very good accuracy and computational efficiency on large-scale, high-dimensional tabular data, making the process of feature interaction modeling quick and reliable. CatBoost improves the model’s performance on a dataset with numerous categorical features by naturally handling them to reduce overfitting and preserve necessary feature associations. In a high-stakes financial decision environment, interpretability is the main thing. EBM represents both individual and pairwise feature contributions, thus making predictions understandable and regulation-compliant. BBOA and PO are two different but complementary metaheuristic optimizers inspired by nature: PO integrates broad search with focused exploitation, whereas BBOA balances intensification and exploration. The insertion of these two to pack enhances not only the adaptation to complex, non-convex solution spaces but also the hyperparameter optimization. Individually, they can provide a solid, interpretable, and efficient system that, when combined, can further strengthen the credit risk analysis to be accurate, feasible, and thorough across various financial datasets.

Table 3 presents a technical overview of the methodological framework that underpins the research, showing how different computational techniques combine to form a powerful pipeline for credit risk prediction. At the core, the architecture tackles the complicated challenges of large-scale financial data by integrating transparent additive modeling, state-of-the-art boosting methods, and metaheuristic optimization. The strong generalization that boosting methods offer on high-dimensional tabular data, along with their compatibility with accelerated training, enable rapid iteration and deployment in data-intensive scenarios. The structural transparency that the interpretable additive model brings ensures that feature-level contributions are still traceable and meet regulatory requirements. Metaheuristic optimizers lessen the possibility of converging to less-than-ideal configurations and explore multimodal, non-convex hyperparameter spaces, thus increasing the flexibility of the system. The synergistic, or mutually helpful, design of the components therefore leads to a technically robust prediction framework capable of generating accurate, stable, and understandable credit risk assessments by integrating interpretability, computational efficiency, and global search capabilities.

**Table 3:** A comprehensive overview of the methods used is presented, highlighting their strengths and contributions to robust, interpretable, and efficient credit risk prediction.

Method/algorithm	Purpose/role	Key strengths	Practical benefit in credit risk prediction
LightGBM	Gradient boosting model	Efficient on high-dimensional tabular data; histogram-based binning; GPU acceleration; leaf-wise tree growth	High accuracy and fast training on large-scale financial datasets
CatBoost	Gradient boosting model	Native handling of categorical features; ordered boosting; reduces prediction shift	Stable generalization, robust handling of categorical financial attributes, minimizes preprocessing
EBM	Interpretable model	GAM-based with pairwise interactions; additive and transparent	Explains feature contributions for regulation-friendly, accountable decision-making
BBOA	Metaheuristic optimizer	Nature-inspired exploration/exploitation; good for complex, multimodal solution spaces	Efficient hyperparameter tuning and alternative classification perspective
PO	Metaheuristic optimizer	Hunting-inspired exploration/exploitation; breaks local optima	Enhances model adaptability and improves hyperparameter optimization in non-convex spaces
Synergistic Design	Combined methodological framework	Leverages complementary strengths of boosting, interpretable modeling, and metaheuristics	Well-rounded, interpretable, efficient, and resilient credit risk prediction framework

### Evaluation metrics

Accuracy is the fraction of correctly classified instances in all the evaluated samples. It indicates the overall performance of the model across all classes. However, despite its popularity, it can be misleading when applied to an unbalanced dataset.

1 $$Accuracy=\frac{TP+TN}{TP+TN+FP+FN}$$

Precision is the measure of the positive instances that were expected and actually turned out to be positive. To be specifically very accurate, only in the case when false positives are the ones that bring the heavy consequences, precision is very critical. Such a model of high precision can yield reliable, low-error outcomes.

2 $$Precision= \frac{TP}{TP+FP}$$

Recall measures the ability of a model to correctly identify all real positive cases. This parameter is very important in situations where missing positive instances could lead to serious consequences. A high recall means the model is effectively reducing the number of false negatives.

3 $$Recall= \frac{TP}{TP+FN}$$

Since the F1 Score is the harmonic mean of precision and recall, it gives a just assessment to both metrics. F1 score is the metric of choice in case of imbalanced class distributions or if there is a need to find a balance between precision and recall. Hence, it is a good indicator of the model’s ability to detect true positives while maintaining a low number of false negatives.

4 $$F1=2 . \frac{Precision . Recall}{Precision+Recall}$$

## Results and discussion

### Model evaluation

To ensure robustness and reproducibility, a stratified five-fold cross-validation strategy was employed. The dataset was first divided into training (80%) and holdout test (20%) sets using stratified sampling. The training set was used exclusively for model development, including preprocessing, feature engineering, and hyperparameter optimization via five-fold cross-validation. All transformations were applied within each fold to prevent data leakage. After selecting the best-performing model, it was retrained on the full training dataset and evaluated on the independent test set. The average performance across all folds was used as the final evaluation metric, while fold-wise results are reported in Table 4 to demonstrate model stability and generalization capability.

**Table 4:** Result of the K-fold cross-validation.

Model	Metric	K1	K2	K3	K4	K5
LGBM	Accuracy	0.8812	0.8876	0.8923	0.8960	0.8894
	F1-Score	0.8835	0.8892	0.8948	0.8981	0.8910
CAT	Accuracy	0.8925	0.8981	0.9034	0.9062	0.8993
	F1-Score	0.8941	0.9002	0.9051	0.9077	0.9015
EMB	Accuracy	0.8723	0.8789	0.8837	0.8864	0.8801
	F1-Score	0.8746	0.8812	0.8861	0.8885	0.8827

The dataset exhibits a moderate class imbalance, with approximately 70% non-default and 30% default instances. In this study, no synthetic oversampling techniques (e.g., SMOTE) were applied to avoid the risk of introducing artificial patterns and potential overfitting. Instead, class imbalance was addressed through a combination of strategies. First, stratified sampling was employed during the train–test split and K-fold cross-validation to preserve the original class distribution across all subsets. Second, cost-sensitive learning was incorporated by assigning higher misclassification penalties to the minority (default) class during model training. Third, model performance was evaluated using imbalance-aware metrics, including F1-score and precision–recall-based measures, rather than relying solely on accuracy. These measures ensure that the models remain sensitive to the minority class while maintaining overall predictive stability. The consistency of performance across folds (Table 4) further confirms that the models are not biased toward the majority class.

### Improving model accuracy through iterative parameter optimization

**Fig. 5:**
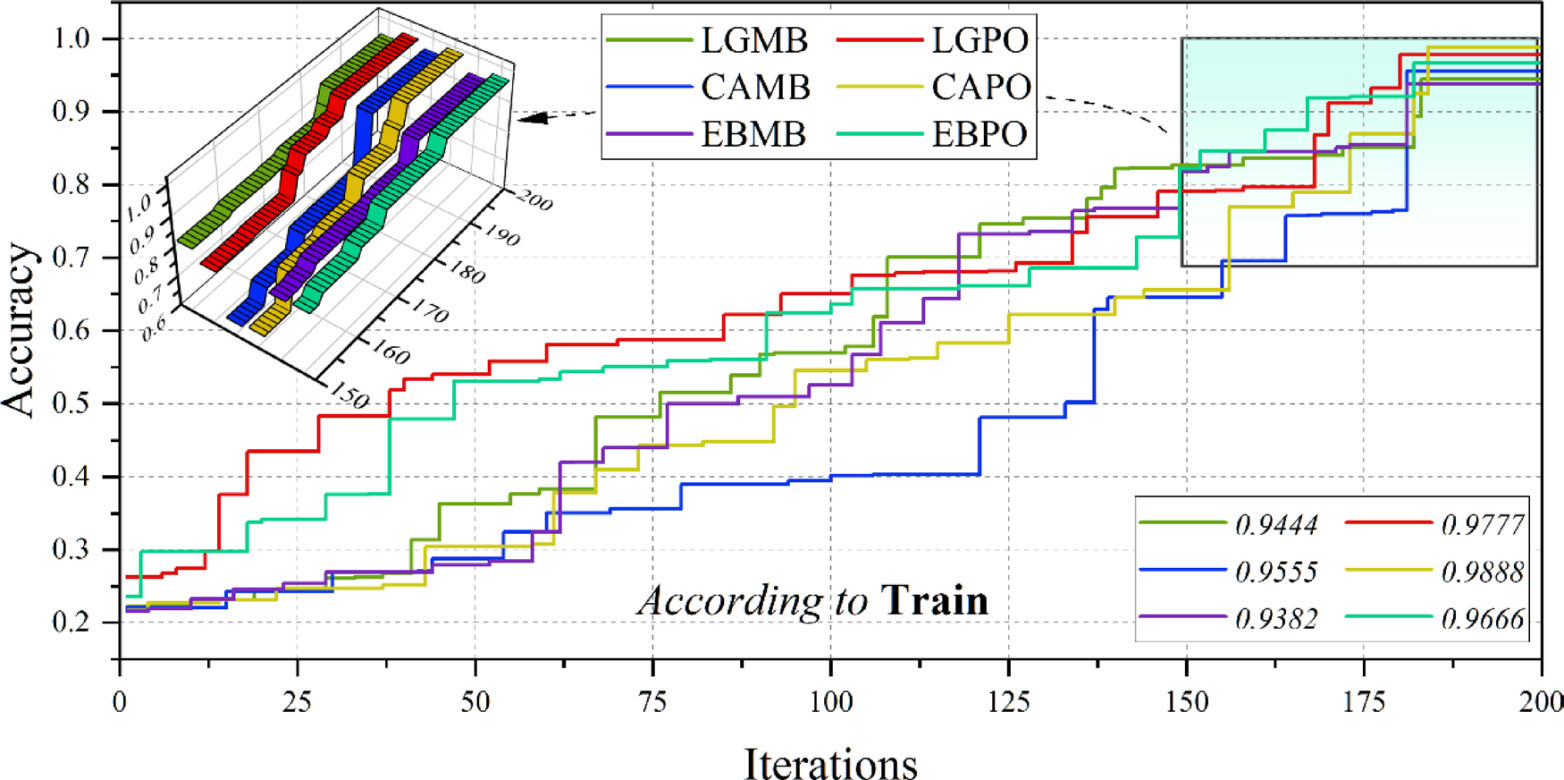
The *convergence* trend of the iterative solution, demonstrating steady improvement and stabilization of the model’s output.

The development of model accuracy through 200 optimization iterations for various methodological setups is depicted in Fig. 5. The gradual improvements of the curves reflect the discrete improvements as the optimization procedure discovers more efficient parameter choices. The convergence zone marked near the last iterations, where all configurations reach high accuracy values, shows the stabilization that indicates successful navigation of the underlying non-convex search space. Such repetitive nature is crucial from a practical point of view for use cases like credit risk assessment, where steady and reliable convergence ensures consistent results under operational constraints. The upward trends signal that with continuous optimization, each configuration becomes more discriminative, thus allowing for more reliable, data-driven financial system decision-making. In mathematical terms, the image is akin to iterative refinement in optimization theory, wherein each step represents a move toward a local or global optimum. One may consider gradient-based and metaheuristic dynamics to account for the increasing accuracy curves, which reflect a trade-off between exploration and exploitation. The system is transitioning from higher-energy (poorly optimized) states to lower-energy stable equilibria, which is a physical analogy of energy reduction processes.

The optimization was conducted over predefined hyperparameter ranges, including number of estimators (20–100), learning rate (0.01–0.1), maximum tree depth (3–10), and subsample ratio (0.6–1.0). Each candidate solution represents a specific combination of these parameters. Both BBOA and PO were implemented with a population size of 20 agents and executed for 200 iterations. During each iteration, candidate solutions were updated according to the respective algorithmic rules to search for optimal configurations.

To enhance reproducibility and clarify the experimental setup, Table 5 summarizes the key hyperparameter values employed for each baseline and optimization-driven model configuration. These parameters were fixed during training to ensure consistent and controlled comparison between baseline and optimized variants. The reported settings represent the final configurations used to generate the performance results discussed in this part.

**Table 5:** Key hyperparameter settings for baseline and optimized models.

Parameter	Models								
	LGBM	LGBB	LGPO	CAT	CABB	CAPO	EBM	EBBB	EBPO
N estimators	20	50	70	–	–	–	–	–	–
Random state	40	33	29	–	–	–	–	–	–
Iterations	–	–	–	20	60	80	–	–	–
Verbose	–	–	–	100	90	70	–	–	–
Learning rate	–	–	–	–	–	–	0.02	0.05	0.06
Random state	–	–	–	–	–	–	39	34	30

Table 6 presents a detailed account of how the different modeling setups have performed during the training, testing, and merged datasets with the use of standard classification metrics. The four metrics of accuracy, precision, recall, and F1 score give a very complete picture of the predictive reliability of each model as well as their ability to generalize beyond the training data. Models that have been improved through optimization regularly show higher test-set performance, thus they are better able to resist overfitting and efficiently navigate the parameter search space. The pronounced improvements of the optimized configurations over the baselines, particularly in the gradient-boosting and interpretable model families, exemplify this behavior. Balanced classification behavior, which can be inferred from the close matching of accuracy and recall values across the different entries, is a very important aspect that is additionally confirmed here. The high scores on the test set, which also point to considerable model stability when faced with unknown data, are thus a further indication of the soundness of the underlying analytical approach. In short, Table 6 demonstrates the effect of systematic optimization in improving the accuracy and operational viability of credit risk categorization by enhancing predictive precision and balancing performance across key variables.

**Table 6:** The performance of the LGB, CAT, and* EBM* models was evaluated using established performance metrics.

Index	Model					
	LGBB			LGPO		
	Train	Test	All	Train	Test	All
Accuracy	0.9444	0.9703	0.9496	0.9778	0.9901	0.9802
Precision	0.9482	0.9703	0.9522	0.9784	0.9904	0.9808
Recall	0.9444	0.9703	0.9496	0.9778	0.9901	0.9802
F1 Score	0.9452	0.9703	0.9502	0.9779	0.9901	0.9803

Index	Model		
	LGBM		
	Train	Test	All
Accuracy	0.8963	0.9158	0.9002
Precision	0.9036	0.9187	0.9065
Recall	0.8963	0.9158	0.9002
F1 Score	0.8981	0.9167	0.9018

Index	Model					
	CABB			CAPO		
	Train	Test	All	Train	Test	All
Accuracy	0.9556	0.9802	0.9605	0.9889	0.9950	0.9901
Precision	0.9578	0.9806	0.9622	0.9891	0.9951	0.9903
Recall	0.9556	0.9802	0.9605	0.9889	0.9950	0.9901
F1 Score	0.9560	0.9803	0.9608	0.9889	0.9951	0.9902

Index	Model		
	CAT		
	Train	Test	All
Accuracy	0.9062	0.9257	0.9101
Precision	0.9127	0.9285	0.9157
Recall	0.9062	0.9257	0.9101
F1 Score	0.9077	0.9265	0.9114

Index	Model					
	EBBB			EBPO		
	Train	Test	All	Train	Test	All
Accuracy	0.9383	0.9455	0.9397	0.9667	0.9851	0.9704
Precision	0.9424	0.9467	0.9431	0.9679	0.9853	0.9713
Recall	0.9383	0.9455	0.9397	0.9667	0.9851	0.9704
F1 Score	0.9391	0.9459	0.9405	0.9669	0.9852	0.9706

Index	Model		
	EBM		
	Train	Test	All
Accuracy	0.8864	0.9059	0.8903
Precision	0.8946	0.9108	0.8978
Recall	0.8864	0.9059	0.8903
F1 Score	0.8885	0.9072	0.8922

A comparison set of confusion matrices illustrating the agreement between real and expected class labels for various modeling scenarios is presented in Table 7. The primary goal of this table is to reveal classification behavior from an open, instance-level perspective by showing not only the number of times each model correctly identifies default and non-default situations, but also the places of misclassifications. In risk-sensitive environments, where false positives and false negatives result in different types of operational and financial consequences, this level of detail is very important for the verification of model reliability. True-negative and true-positive values that are higher signal that better variants of the models demonstrate a substantial decrease in the number of misclassifications. For example, configurations such as LGPO, CAPO, and EBPO considerably lower false-positive and false-negative rates, indicating that the models better differentiate between borrowers who fail and those who do not. These improvements refer to enhanced generalization capacity and more efficient decision boundaries. Consequently, the table functions as a diagnostic instrument that reveals the detailed effects of optimization on model behavior. It enables more informed evaluations of risk management effectiveness by focusing on the balance between correct and incorrect predictions and highlighting the real-world benefits of the improved methodology design.

**Table 7:** Confusion Matrix Summarizing Predicted and Actual Class Labels.

Models		Default	
	Default	No	Yes
LGBM	No	629	71
	Yes	30	282
LGBB	No	661	39
	Yes	12	300
LGPO	No	684	16
	Yes	4	308
CAT	No	635	65
	Yes	26	286
CABB	No	669	31
	Yes	9	303
CAPO	No	691	9
	Yes	1	311
EBM	No	622	78
	Yes	33	279
EBBB	No	654	46
	Yes	15	297
EBPO	No	677	23
	Yes	7	305

The high predictive performance achieved by optimized configurations is attributed to rigorous data preprocessing, controlled resampling, strict train–test separation, and the use of metaheuristic optimization for hyperparameter tuning. Balanced precision, recall, and F1 scores, together with confusion matrix analysis, indicate stable generalization rather than overfitting or data leakage.

Baseline models exhibit performance consistent with prior studies, while optimization-driven improvements highlight the benefit of advanced global search strategies.

### Quantifying model differences and feature contributions in predictive analytics

Table 8 shows a detailed comparison of model performance, highlighting significant and subtle differences across configurations and providing a basis for informed selection. The primary objective of this analysis is to discern which models differ significantly in performance and which exhibit similar behavior, thereby providing a firm basis for prudent model selection. The findings reveal intricate patterns within the different model families. The predictive outcomes vary depending on algorithmic configurations, as evidenced by substantial performance disparities between some LightGBM settings and models from the CAT and EBM families. Furthermore, divergences in the CAT family range from significant to almost non-existent, indicating that the choice of the model can be influenced by factors such as interpretability, computational efficiency, or implementation difficulty in those regions. The fluctuation of ensemble models, in particular, underscores the role of model architecture and hyperparameterization in prediction accuracy, as some configurations can be better or worse than others. Moreover, cross-family comparisons illuminate the performance landscape by identifying pairs of models whose performance can hardly be distinguished and those that are statistically different.

**Table 8:** Result of the Wilcoxon test.

Difference between models	Parameter		Difference between models	Parameter	
	statistic	p_value		statistic	p_value
LGBM vs LGBB	459	0.0477	LGPO vs EBM	1334	0.0005
LGBM vs LGPO	1066	0.0013	LGPO vs EBBB	231	0.0030
LGBM vs CAT	22	0.5271	LGPO vs EBPO	16.5	0.2059
LGBM vs CABB	651	0.0150	CAT vs CABB	442	0.0173
LGBM vs CAPO	1334	0.0005	CAT vs CAPO	1025	0.0006
LGBM vs EBM	16.5	0.2059	CAT vs EBM	73.5	0.1797
LGBM vs EBBB	307.5	0.1138	CAT vs EBBB	170.5	0.1441
LGBM vs EBPO	828	0.0030	CAT vs EBPO	589	0.0032
LGBB vs LGPO	128	0.0071	CABB vs CAPO	124	0.0106
LGBB vs CAT	287	0.0578	CABB vs EBM	864	0.0063
LGBB vs CABB	18	0.1317	CABB vs EBBB	66	0.0495
LGBB vs CAPO	231	0.0030	CABB vs EBPO	11	0.0578
LGBB vs EBM	640.5	0.0201	CAPO vs EBM	1632	0.0002
LGBB vs EBBB	16.5	0.2059	CAPO vs EBBB	364	0.0013
LGBB vs EBPO	55	0.0164	CAPO vs EBPO	63	0.0736
LGPO vs CAT	792	0.0014	EBM vs EBBB	459	0.0477
LGPO vs CABB	52.5	0.0253	EBM vs EBPO	1066	0.0013
LGPO vs CAPO	16.5	0.2059	EBBB vs EBPO	128	0.0071

**Fig. 6:**
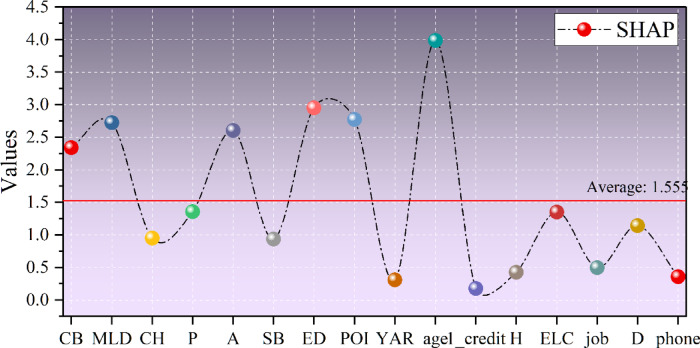
SHAP values quantifying the marginal influence of each feature on prediction behavior.

The predictive SHAP (SHapley Additive exPlanations) model (Fig. 6) helps in understanding the flow of each parameter towards the model’s output. The points in the plot represent every instance, which is a feature value in the data set. The components are on the x-axis, and the corresponding values are on the y-axis. Each component has a horizontal red line that denotes the data’s average. The diameter of the disc and the color of the point represent the length and the sign of the effect, respectively, i.e., whether the variable adds or subtracts from the prediction. The peaks and valleys of the function portray the parts of the variables and their positive and negative effects. The picture represents the SHAP values of the different attributes that were used in a credit risk prediction model. Each number indicates a feature that contributes to the model’s output. Since features with a higher SHAP value have a greater impact on the expected credit risk, a modification in these features has a major effect on the model’s choices. For example, the feature “agel_credit” has been singled out as having the highest (~ 4.0) value, indicating that it is the most influential feature in risk assessment and may also indicate a key financial or demographic factor, such as credit age or length of credit history. Features with lower SHAP values, such as “phone” and “job”, have lesser contributions to the prediction, signifying that their effect on the outcome is more indirect or subtle. The horizontal red line depicting the average SHAP value (~ 1.55) helps in deciding whether the traits are above or below average in importance. The model decides which attributes are more predictive by their ability to explain changes in the target variable (credit risk), which causes fluctuations in SHAP values. These major features may be essential borrower traits or financial habits that are highly correlated with default risk. Less significant features may be less predictive or redundant due to their association with more dominant qualities.

#### Scope of model comparison

The comparative structure of this study is intentionally designed as a controlled within-family evaluation. Baseline and optimized versions of the same model architectures are compared in order to isolate the effect of metaheuristic optimization on model performance, stability, and interpretability. This approach avoids confounding influences arising from fundamentally different algorithmic structures. Models and tuning strategies reported in prior literature (e.g., XGBoost, grid search, and random search methods) are cited to contextualize the work within the broader research landscape, rather than to serve as direct experimental baselines. Accordingly, the contribution of this study is positioned as a methodological analysis of optimization-driven learning, not as an exhaustive inter-algorithm benchmarking study.

From a business perspective, recall is critical because it reflects the model’s ability to correctly identify high-risk borrowers. A low recall would result in missed default cases, potentially leading to increased financial losses for lenders. On the other hand, precision indicates the proportion of correctly identified non-default cases among all predicted non-defaults. High precision reduces the likelihood of false rejections, thereby improving customer acceptance rates and reducing opportunity costs. Therefore, the high F1-score achieved by the proposed model indicates a balanced trade-off between minimizing credit risk exposure and maintaining efficient approval rates, which is essential in practical digital lending scenarios.

### Application in real-world

This section presents a conceptual and scenario-based discussion of the potential real-world relevance of the proposed models. The analysis is not based on deployed pilot projects or operational case studies, but instead extrapolates practical implications from the experimental results, model stability, and interpretability analyses presented in the preceding sections. Accordingly, references to operational benefits should be interpreted as indicative outcomes rather than empirically validated field measurements.

#### Operational relevance

The optimized predictive models generated through repeated parameter refinements have a significant impact across various risk-sensitive fields, such as credit evaluation, financial risk management, and regulatory compliance, among others. The main risk in an experiment is the inability to discriminate between between scenarios. In this case, the power of generalization and the accuracy are the factors leading to a reduction of operational risk and financial loss. Iterative optimization is the link to operational deployment when consistency issues arise, as it ensures stability and reliability in real-life situations.

#### Interpretability and feature significance

Along with their basic prediction performance, these models also offer locally understandable explanations of the feature-level contributions. Being interpretable, users can identify the most influential factors and even list them in order, which helps them pinpoint interventions, policy changes, and the efficient use of resources. Transparency in decision-making is enhanced by understanding the features that affect model predictions, and thus predictive analytics remain consistent with real business goals.

#### Cross-domain applicability and strategic impact

Being methodically optimized and interpretable, these models are broadly applicable outside the financial industry, for instance, in healthcare, insurance, and engineering sectors, where exact, clear, and stable predictions are indispensable. The models equip companies with tools to reduce risks arising from their operations, make better decisions in the changing real-world environment, and turn complex data into understandable insights by combining near-accurate predictions of the future with interpretative clarity.

**Fig. 7:**
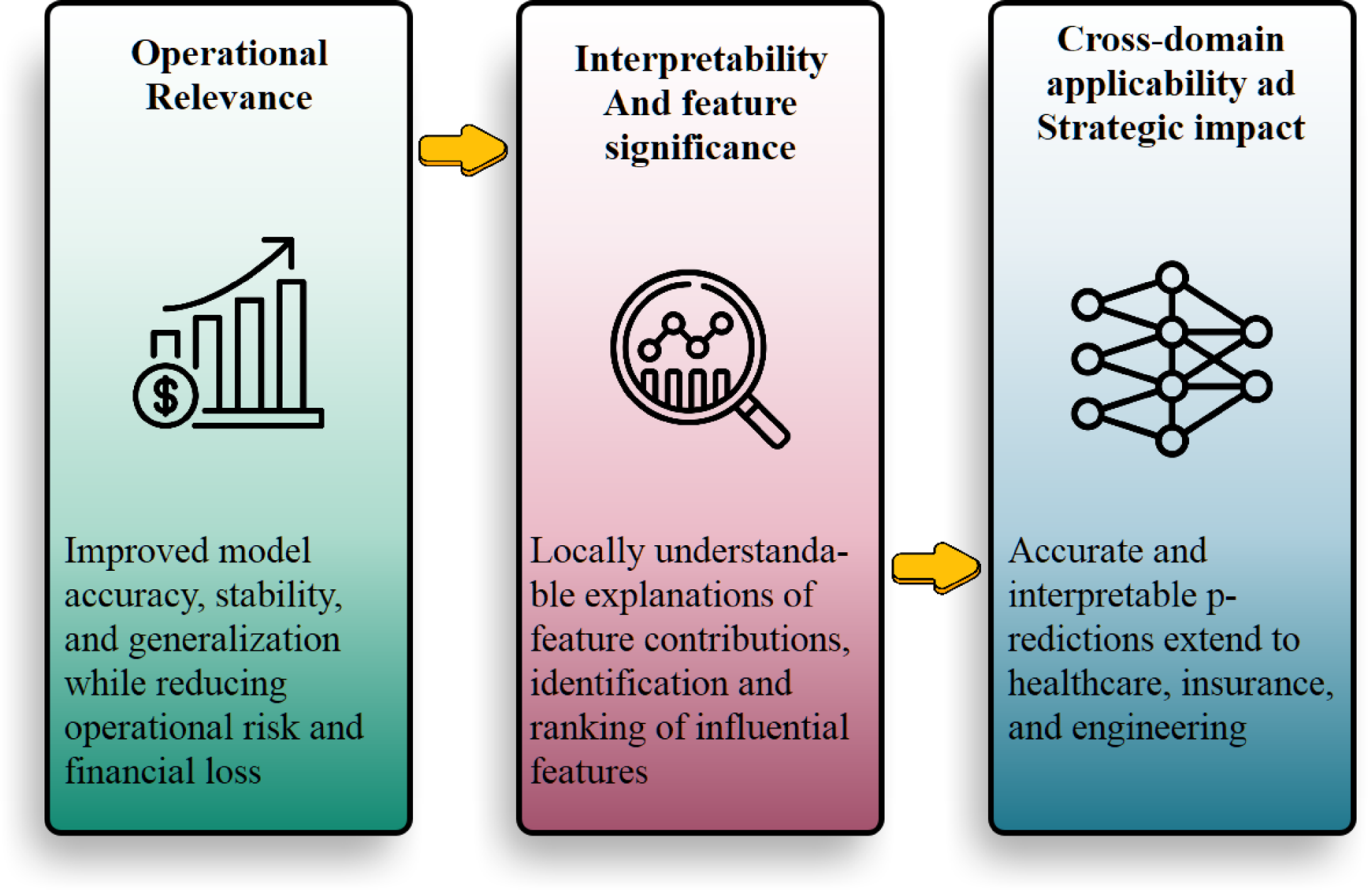
Predictive modeling progression from operational relevance to strategic impact.

Figure 7 presents a three-pillar flow illustrating the evolution of predictive modeling from technical performance to broader strategic usefulness. The first pillar, Operational Relevance, shows how a robust model design reduces operational and financial risk through better accuracy, stability, and generalization. The second pillar, Interpretability and Feature Significance, leverages the first one by stressing the importance of concise, locally understandable explanations that highlight feature contributions and point out the factors. The third pillar, Cross-domain Applicability and Strategic Impact, describes how precise and understandable models can be used in a variety of industries such as engineering, healthcare, and insurance. Therefore, these pillars convey a powerful message: a substantial AI effect occurs when strong performance, clear interpretability, and domain diversity are combined to create reliable, high-value prediction systems.

## Conclusion

The study reveals the potential of nature-inspired metaheuristic optimization, combined with advanced machine learning techniques, for credit risk prediction, especially for underbanked and non-traditional borrowers. The rise of buy now, pay later (BNPL) and other alternative financing methods has, in fact, exposed the limitations of conventional credit scoring models that are often incapable of capturing complex financial, demographic, and behavioral patterns. By a hybridization of Brown Bear Optimization Algorithm (BBOA) and Puma Optimizer (PO) with ensemble gradient boosting models, i.e., LightGBM, CatBoost, and Explainable Boosting Machine (EBM), the proposed framework achieves significant operational stability, generalization, and predictive accuracy improvements under different high-dimensional datasets. The methodological approach simultaneously resolves transparency and trust issues by ensuring that the prediction system is not only interpretable but also performant in the case of high-stakes financial decision-making. Feature-level analyses, such as SHAP and permutation importance, reveal default risk factors closest to the core, thus providing risk managers, policymakers, and lending institutions with the insights of greatest value. This degree of interpretability, on the one hand, is necessary to build user trust in automated credit evaluation systems and, on the other hand, is a requirement for the regulatory framework. In fact, the iterative optimization process, which is the main reason, among other things, for their ability to handle the nonlinear interactions and data imbalances typical of real-world financial datasets, is the origin of their robustness.

This research delves significantly deeper than standard credit scoring methods and has the potential to change the world in a very positive way. Banks could employ transparent machine learning models alongside hybrid optimization procedures to not only minimize portfolio risks but also raise credit limits and create policies that encourage financial inclusion. From a product standpoint, the models facilitate the rollout of lending practices backed by solid evidence, making it harder for the institution to suffer financial losses, as well as decreasing the occurrence of misclassification errors. By offering a sturdy, transparent prediction framework, the paper paves the way for the digital finance sector to adopt more responsible, informed decision-making processes, which are in flux due to rapid change. Moreover, the framework can be taken to a new level by the eventual inclusion of multi-objective optimization, adaptive real-time learning strategies, and complex deep learning architectures. Furthermore, in a regulated environment where there is a need for AI-powered credit evaluation, coupling optimization-driven models with sophisticated explainability techniques may enhance auditability and accessibility. Simply put, these various ways can, on the one hand, substantially deepe n the scientific understanding of the phenomena and, on the other hand, markedly facilitate the practical application of the systems that predict credit risk, thus, effectively, consolidating these instrumental tools as a firm part of risk management, a crucial factor in going e, and a helpful agent of long-term growth in modern financial ecosystems.

## Data Availability

Data will be provided upon reasonable request by the corresponding author.

## References

[1] De Silva, C. Advancing financial risk management: AI-powered credit risk assessment through financial feature analysis and human-centric decision-making. **25**(1), 1775–1785 (2025).

[2] Zhou, G. & Wang, S. Enhancing credit risk decision-making in supply chain finance with interpretable machine learning model. *IEEE Access* **13**, 14239–14251. 10.1109/ACCESS.2025.3530433 (2025).

[3] Zhang, D. & Ma, X. Machine learning-based credit risk assessment for green bonds: Climate factor integration and default prediction analysis. *J. Sustain. Policy. Pract.* **1**(2), 121–135 (2025).

[4] Maddodi, B. S., Shwetha, V., Nirmala, R. & Gopika, S. V. Advances and challenges in Artificial Intelligence-driven flood and drought risk management: A comprehensive review. *Eng. Appl. Artif. Intell.* **164**, 113354 (2026).

[5] Han, X., Yang, Y., Chen, J., Wang, M. & Zhou, M. Symmetry-aware credit risk modeling: A deep learning framework exploiting financial data balance and invariance.. *Symmetry*10.3390/sym17030341 (2025).

[6] Sagoe, A. A. & Ashun, A. K. Influences of credit risk management on financial resilience of SMEs in Ghana. *Asian J. Econ. Bus. Account.* **25**(11), 506–525 (2025).

[7] Aruleba, I. & Sun, Y. An improved ensemble method with data resampling for credit risk prediction. *IEEE Access* **13**, 71275–71287. 10.1109/ACCESS.2025.3563432 (2025).

[8] S. Joshi, “Gen AI for market risk and credit risk learn agentically powered gen AI; gen AI agentic framework for financial risk management,” *Gen AI Agentic Framew. Financ. Risk Manag. (January 15, 2025)*, 2025.

[9] Zhu, Y. & Wu, D. P2P credit risk management with KG-GNN: A knowledge graph and graph neural network-based approach. *J. Oper. Res. Soc.* **76**(5), 866–880 (2025).

[10] Machado, M. R., Chen, D. T. & Osterrieder, J. R. An analytical approach to credit risk assessment using machine learning models. *Decis. Anal. J.* **16**, 100605. 10.1016/j.dajour.2025.100605 (2025).

[11] Shang, L., Zhao, J., Li, G. & Zhang, X. Survival analysis in credit risk management: A review study. *J. Credit Risk* **20**(4), 59–83 (2025).

[12] Chew, J., Shen, Z., Hu, K., Wang, Y. & Wang, Z. Artificial intelligence optimizes the accounting data integration and financial risk assessment model of the e-commerce platform. *Int. J. Manag. Sci. Res.* **8**(2), 7–17 (2025).

[13] Lin, S., Song, D., Cao, B., Gu, X. & Li, J. Credit risk assessment of automobile loans using machine learning-based SHapley Additive exPlanations approach. *Eng. Appl. Artif. Intell.* **147**, 110236 (2025).

[14] Lu, Y., Yang, L., Shi, B., Li, J. & Abedin, M. Z. A novel framework of credit risk feature selection for SMEs during industry 4.0. *Ann. Oper. Res.* **350**(2), 425–452 (2025). 10.1007/s10479-022-04849-3 PMC9309243 35910041

[15] Qi, R. An empirical study on credit risk assessment using machine learning: Evidence from the Kaggle credit card fraud detection dataset. *J. Comput. Signal Syst. Res.* **2**(5), 48–64 (2025).

[16] Munerman, I. AI and finance: Redefining risk analysis through advanced technologies. In *Decoding AI*, 120–130 (Productivity Press, 2026).

[17] Löschenbrand, S., Maier, M., Millischer, L. & Resch, F. Credit risk where it’s due (2025).

[18] Karami, A. & Igbokwe, C. The impact of big data characteristics on credit risk assessment. *Int. J. Data Sci. Anal.* **20**, 1–21 (2025).

[19] Roy, J. K. & Vasa, L. Transforming credit risk assessment: A systematic review of AI and machine learning applications. *Journal of Infrastructure Policy and Development* **9**(1), 9652 (2025).

[20] Chafale, J. H., Wadetwar, R. S., Giriya, D. M., Badhiye, S., Borkar, P. & Shinde, S. Credit risk analysis using machine learning. In *2024 8th International Conference on Computing, Communication, Control and Automation (ICCUBEA)*, 1–5 (2024).

[21] Papa, J. F. & Ricafort, R. B. Analysis on credit risk assessment for a multi-purpose cooperative using neural network algorithm.

[22] Soni, D., Singh, K., Choudhary, A. & Pathak, D. K. Machine learning-based credit risk prediction: A systematic review of techniques, challenges, and future directions. *Int. J. Res. Rev. Appl. Sci. Humanit. Technol.* (2025).

[23] Muhindo, J., Mukasa, K., Kitakufe, D. & Kato, J. Advancing credit risk assessment and financial decision-making: Integrating modern techniques and insights. *World J. Adv. Res. Rev.* **23**(2), 2019–2027 (2024).

[24] Ke et al. G. Lightgbm: A highly efficient gradient boosting decision tree. *Adv. Neural Inf. Process. Syst.* **30** (2017).

[25] Wang, D., Zhang, Y. & Zhao, Y. LightGBM: An effective miRNA classification method in breast cancer patients. In *Proceedings of the 2017 International Conference on Computational Biology and Bioinformatics*, 7–11 (2017).

[26] Hancock, J. T. & Khoshgoftaar, T. M. CatBoost for big data: An interdisciplinary review. *J. big data* **7**(1), 94 (2020). 33169094 10.1186/s40537-020-00369-8 PMC7610170

[27] Prokhorenkova, L., Gusev, G., Vorobev, A., Dorogush, A. V. & Gulin, A. “CatBoost: Unbiased boosting with categorical features. *Adv. Neural Inf. Process. Syst.* **31** (2018).

[28] Liu, G. & Sun, B. Concrete compressive strength prediction using an explainable boosting machine model. *Case Stud. Constr. Mater.* **18**, e01845 (2023).

[29] Sarica, A., Quattrone, A. & Quattrone, A. Explainable boosting machine for predicting Alzheimer’s disease from MRI hippocampal subfields. In *International Conference on Brain Informatics*, 341–350 (2021).

[30] Prakash, T., Singh, P. P., Singh, V. P. & Singh, S. N. A novel brown-bear optimization algorithm for solving economic dispatch problem. In *Advanced Control & Optimization Paradigms for Energy System Operation and Management* 137–164 (River Publishers, 2023).

[31] Abdollahzadeh B. et al. Puma optimizer (PO): A novel metaheuristic optimization algorithm and its application in machine learning. *Cluster Comput.* 1–49, (2024).

[32] Maurya, P., Tiwari, P. & Pratap, A. Puma optimizer technique for optimal planning of different types of distributed generation units in radial distribution network considering different load models. *Electr. Eng.* **107**(3), 2777–2828 (2025).

